# Urinary orosomucoid and accelerated GFR decline: a 11-year follow-up study in a non-diabetic population

**DOI:** 10.1093/ndt/gfaf113

**Published:** 2025-07-04

**Authors:** Runa M Andreassen, Karl M Brobak, Inger Therese Enoksen, Toralf Melsom, Bjørn O Eriksen, Marit D Solbu

**Affiliations:** Department of Internal Medicine, Helgeland Hospital Trust, Sandnessjøen, Norway; Metabolic and Renal Research Group, UiT, The Arctic University of Norway, Tromsø, Norway; Section of Nephrology, University Hospital of North Norway, Tromsø, Norway; Metabolic and Renal Research Group, UiT, The Arctic University of Norway, Tromsø, Norway; Section of Nephrology, University Hospital of North Norway, Tromsø, Norway; Metabolic and Renal Research Group, UiT, The Arctic University of Norway, Tromsø, Norway; Metabolic and Renal Research Group, UiT, The Arctic University of Norway, Tromsø, Norway; Section of Nephrology, University Hospital of North Norway, Tromsø, Norway; Metabolic and Renal Research Group, UiT, The Arctic University of Norway, Tromsø, Norway; Section of Nephrology, University Hospital of North Norway, Tromsø, Norway; Metabolic and Renal Research Group, UiT, The Arctic University of Norway, Tromsø, Norway; Section of Nephrology, University Hospital of North Norway, Tromsø, Norway

To the Editor,

Traditional risk markers for chronic kidney disease (CKD) have limited sensitivity in detecting the disease at an early stage, making the search for novel biomarkers essential [1]. Urinary orosomucoid excretion (UOE) has been linked to generalized endothelial dysfunction, atherosclerosis and renal impairment among patients with diabetes [2]. However, it remains unclear whether this association extends to the general population. Our study aimed to explore the relationship between UOE, an early marker of endothelial dysfunction, and the decline in glomerular filtration rate (GFR) within a middle-aged general population cohort.

The Tromsø study is a population-based, prospective study with repeated health surveys of the inhabitants of Tromsø, a municipality in Northern Norway [3]. As part of Tromsø6 (2007–08), the Renal Iohexol Clearance Survey (RENIS-T6, 2007–09) invited all participants between 50 and 62 years of age without diabetes or self-reported cardiovascular or renal disease, and GFR was measured using single-sample plasma iohexol clearance [4]. Follow-up studies were performed in 2013–15 (RENIS-FU) and 2018–20 (RENIS-3). Urinary specimens were collected during Tromsø6 from three consecutive days. Urinary albumin and creatinine concentrations were analyzed immediately, while UOE was subsequently analyzed in Denmark. Urinary albumin–creatinine and orosomucoid–creatinine ratios (UOCR and UACR) were calculated for each sample, and median values were used. The 2012 Chronic Kidney Disease Epidemiology Collaboration equation was used to calculate estimated GFR based on creatinine and cystatin C (eGFRcrecys) [5].

To evaluate the associations between the urinary markers and the annual change in GFR, we used a linear mixed model with random intercepts and slopes for time. The association between accelerated GFR decline, defined as the 10% of all subjects with the steepest GFR decline slope, was assessed using multivariable logistic regression. To quantitate the contribution of UOCR above vs equal to/below the median to the prediction of accelerated GFR decline, we calculated the area under the receiver-operating characteristics (ROC) curve (AUC) for the nested logistic regression model. The supplementary data describe the different methods and models in more detail.

A total of 1589 participants were included, providing 4323 GFR measurements, with a mean follow-up time of 11.3 (±0.6) years. Supplementary data, Table S1 shows baseline characteristics for our study population. The mean (standard deviation) GFR change was –1.07 (0.03) mL/min/1.73 m²/year, calculated using a linear mixed model (a negative number signifies a decline in GFR). A doubling of UOCR was associated with a faster annual GFR decline of –0.06 (–0.10 to –0.01) mL/min/1.73 m^2^ per year (*P* = .02) when adjusting for age, sex, baseline GFR and UACR. Similarly, UOCR above vs equal to/below the median was associated with a steeper annual GFR decline rate of –0.17 (–0.30 to –0.04) mL/min/1.73 m^2^ (*P* = .008) (Table 1). In the fully adjusted model, UOCR above the median was still associated with a steeper annual decline in GFR [–0.13 (–0.26 to –0.003) mL/min/1.73 m^2^, *P* = .04]. Similar analyses were conducted for UACR, but no significant associations were found with the annual change in GFR, in either crude or adjusted models.

**Table 1:** Change in annual GFR decline explained by urinary markers during the whole study period.

	Doubling (log2) of UOCR		Doubling (log2) of UACR		UOCR above median		UACR above median	
	mL/min/1.73 m^2^/year	*P*-value	mL/min/1.73 m^2^/year	*P*-value	mL/min/1.73 m^2^/year	*P*-value	mL/min/1.73 m^2^/year	*P*-value
Model 1	–0.06 (–0.10 to –0.02),	.01	–0.03 (–0.08 to 0.01),	.15	–0.17 (–0.29 to –0.05),	.01	0.01 (–0.11 to 0.13),	.84
Model 2	–0.06 (–0.11 to –0.02),	.008	–0.03 (–0.08 to 0.02),	.23	–0.16 (–0.27 to –0.05),	.004	0.02 (–0.10 to 0.15),	.74
Model 3	–0.06 (–0.10 to –0.01),	.02	–0.02 (–0.07 to 0.03),	.40	–0.17 (–0.30 to –0.04),	.008	0.03 (–0.09 to 0.16),	.62
Model 4	–0.04 (–0.09 to 0.0007)	.08	–0.01 (–0.06 to 0.05)	.84	–0.13 (–0.26 to –0.003)	.04	0.07 (–0.06 to 0.19)	.28

Model 1: crude, urinary protein (mg/mmol) only.

Model 2: adjusted for sex, age and baseline GFR.

Model 3: adjusted for sex, age, baseline GFR and UACR/UOCR.

Model 4: as in Model 3 and adjusted for hypertension, smoking, glucose, BMI and cholesterol.

GFR: glomerular filtration rate measured with iohexol clearance; hypertension: systolic blood pressure ≥140 mmHg and/or diastolic blood pressure ≥90 mmHg, and/or the use of blood pressure lowering medication.

Accelerated GFR decline corresponded to a mean GFR decline ≥1.53 mL/min/1.73 m²/year (*n* = 159). With the full adjustment, UOCR dichotomized into above vs equal to/below median remained significantly associated with accelerated GFR decline [odds ratio (OR) 1.52, 95% confidence interval (CI) 1.02–2.27, *P* = .04]. UACR was not associated with an accelerated GFR decline in adjusted models (Table 2).

**Table 2:** OR for accelerated GFR decline^a^.

	UOCR			UACR		
	OR	95% CI	*P*-value	OR	95% CI	*P*-value
**Model 1**						
Doubling of urinary protein (log2)	1.23	1.11–1.37	<.001	1.14	1.01–1.28	.03
First quartile	Ref.			Ref.		
Second quartile	0.88	0.51–1.54	.66	0.94	0.58–1.52	.80
Third quartile	1.88	1.15–3.06	.01	0.89	0.55–1.44	.63
Fourth quartile	2.09	1.30–3.37	.002	1.34	0.85–2.10	.20
Equal to/below median	Ref.			Ref.		
Above median	2.11	1.49–2.98	<.001	1.14	0.82–1.58	.44
**Model 2**						
Doubling of urinary protein (log2)	1.20	1.07–1.34	.002	1.14	1.01–1.29	.04
First quartile	Ref.			Ref.		
Second quartile	0.82	0.46–1.46	.49	1.11	0.67–1.87	.68
Third quartile	1.78	1.06–2.96	.03	0.96	0.58–1.61	.88
Fourth quartile	1.84	1.11–3.04	.02	1.50	0.92–2.42	.10
Equal to/below median	Ref.			Ref.		
Above median	2.0	1.39–2.87	<.001	1.15	0.81–1.63	.43
**Model 3**						
Doubling of urinary protein (log2)	1.19	1.05–1.35	.006	1.10	0.97–1.26	.14
First quartile	Ref.			Ref.		
Second quartile	0.81	0.45–1.45	.48	1.10	0.66–1.84	.71
Third quartile	1.76	1.05–2.93	.03	0.95	0.57–1.58	.83
Fourth quartile	1.76	1.05–2.94	.03	1.40	0.86–2.29	.18
Equal to/below median	Ref.			Ref.		
Above median	1.95	1.35–2.82	<.001	1.11	0.78–1.57	.58
**Model 4**						
Doubling of urinary protein (log2)	1.11	0.97–1.28	.14	0.97	0.85–1.12	.69
First quartile	Ref.			Ref.		
Second quartile	0.66	0.36–1.24	.20	1.08	0.61–1.88	.80
Third quartile	1.27	0.73–2.21	.40	0.93	0.54–1.62	.80
Fourth quartile	1.21	0.69–2.11	.51	0.96	0.56–1.66	.90
Equal to/below median	Ref.			Ref.		
Above median	1.52	1.02–2.27	.04	0.91	0.62–1.34	.65

Model 1: crude, urinary protein (mg/mmol) only.

Model 2: adjusted for sex, age and baseline GFR.

Model 3: adjusted for sex, age, baseline GFR and UACR/UOCR.

Model 4: as in Model 3 and adjusted for hypertension, smoke, glucose, BMI, cholesterol.

a Defined as the 10% with steepest decline, annual GFR decline ≥1.53 mL/min/1.73 m^2^.

GFR: glomerular filtration rate measured with iohexol; hypertension: systolic blood pressure ≥140 mmHg and/or diastolic blood pressure ≥90 mmHg, and/or the use of blood pressure lowering medication.

In a model adjusted for the same variables as in the Kidney Failure Risk Equation [6], the AUC of the ROC curve increased from 0.739 to 0.745 (*P* = .002). In the fully adjusted model (including hypertension, body mass index (BMI), cholesterol, glucose and cigarettes per day), AUC increased from 0.831 to 0.832 (*P* = .04) (Supplementary data, Table S4).

Substituting measured GFR with eGFRcrecys as the dependent variable yielded similar results, but the UOCR above versus equal to/below the median was not significantly associated with eGFRcrecys decline in the fully adjusted model (*P* = .056), Supplementary data, Table S6. Accelerated eGFRcrecys decline was defined as a mean annual decline rate ≥2.05 mL/min/1.73 m^2^. Significant associations were observed with OR 1.76 (1.20–2.58; *P* = .004) for accelerated eGFRcrecys decline if UOCR was above the median (Supplementary data, Table S7).

To our knowledge, no previous studies have investigated the association of UOCR with GFR decline in the general population. In a representative sample of relatively healthy middle-aged individuals, we found independent associations between higher baseline levels of UOCR and steeper annual GFR decline rate and increased risk of accelerated GFR decline. Creatinine measurements in blood and albuminuria are currently used to detect and define CKD. A substantial alteration in kidney function must occur to detect a change in eGFR, and not all patients with early kidney damage demonstrate increased UACR. The early stage of kidney disease is usually without symptoms, but if detected, disease progression can be prevented. Our results are in line with several reports indicating that UOCR may serve as an earlier and more sensitive marker for endothelial dysfunction than UACR [2, 7, 8].

The strength of this study was the long follow-up with repeated accurate measurements of GFR in a well-described middle-aged cohort representative of the general non-diabetic population. Sensitivity analysis using eGFRcrecys, which is more frequently assessed in clinical situations, confirmed the association for higher UOCR.

Some limitations have to be addressed. First, this study was observational, and conclusions regarding causality cannot be drawn. Our cohort consisted of middle-aged participants from Northern Europe without diabetes and cardiovascular disease, and the effect size of our findings was relatively small. Thus, whether our results are generalizable across ethnicities and age groups, as well as to patient groups with higher cardiovascular and renal risk, and whether they are clinically relevant, need to be demonstrated. Finally, in our study, UOCR was measured at baseline only, and whether a change in UOCR over time affects kidney function measurements remains to be demonstrated.

To conclude, we demonstrated that a higher level of baseline UOCR was independently associated with a steeper mean annual GFR decline over 11 years using iohexol clearance measurements and GFR estimates, as well as increased risk of accelerated GFR decline, in a general non-diabetic population. Thus, UOCR may be a candidate biomarker for early kidney damage.

## Supplementary Material

gfaf113_Supplemental_File
