# Supplementary material for: Urinary orosomucoid and accelerated GFR decline: a 11-year follow-up study in a non-diabetic population
Source: Nephrol Dial Transplant. 2025 Jul 4;40(11):2210–2. doi: 10.1093/ndt/gfaf113 (PMC12559789; doi:10.1093/ndt/gfaf113)

Table of Contents

Supplementary Methods

Iohexol clearance.. 2

Urinary measurements 2

Other measurements.. 2

Statistical analysis.. 3

Results

Cohort characteristics.. 4

GFR change rates... 4

Accelerated GFR decline 5

Sensitivity analysis.. 5

References.. 6

Supplementary Table 1:

Study population characteristics.. 7

Supplementary Table 2:

Study population characteristics at baseline, lost to follow up.................... 9

Supplementary Table 3:

Prediction of accelerated GFR decline with UOCR above median.............. 10

Supplementary Table 4:

Correlation between urinary markers and covariates................................. 11

Supplementary Table 5:

Change in annual eGFRcrecys decline explained by urinary markers......... 12

Supplementary Table 7:

OR for accelerated eGFRcrecys 13

Supplementary Figure 1:

Flowchart of the study Population.. 14

Supplementary Figure 2:

The non-linear association 15

Supplementary Methods

Iohexol clearance

GFR was measured using single sample plasma iohexol clearance. In short, after an overnight fast, including abstinence from nicotine, 5 ml of iohexol (Omnipaque®, 300mgI/ml, Amersham Health, London, United Kingdom) was injected in a Teflon catheter in an antecubital vein (1). Jacobson's method (2) was used to calculate the optimal time for the single sample, and the minimum sampling time was set to 180 min. The concentration of iohexol was measured by high-performance liquid chromatography in RENIS-T6 and RENIS-FU, and liquid chromatography-mass spectrometry in RENIS-3. The iohexol measurements between RENIS-FU and RENIS-3 were calibrated as previously described (1, 3).

Urinary measurements

Urinary specimens were collected during Tromsø6, a median of 3.8 (IQR 2.4-4.7) months before RENIS-T6. The participants delivered morning urine samples from three consecutive days. The urinary albumin and creatinine concentrations were analysed immediately within 20 hours (autoanalyser ABX Pentra-Horiba and kits from ABX Diagnostics (Montepellier, France). Urinary specimens were frozen at -20℃, and urinary orosomucoid was analysed in Denmark (Randers) by a highly sensitive EU (Europropium)-fluoroimmunosorbent assays using a sandwich technique. Stability studies of urinary orosomucoid have shown stable values despite several freeze-thaw cycles (4).The method (DELFIA- dissociation-enhanced-lanthanide-fluorescence- immunoassay) has been described in more detail previously (5, 6). Urinary albumin and orosomucoid concentrations were divided by the creatinine concentration of the same specimen. Specimens with undetectable urinary albumin were assigned a value of 1 mg/L. The median values of the three samples were used, both for urinary orosomucoid creatinine ratio (UOCR) and urinary albumin creatinine ratio (UACR).

Other measurements

Self-administrated questionnaires were used to collect data about medication, various health issues and smoking habits. Smoking habits were defined as current smokers and the number of cigarettes smoked per day versus never or previous smokers. Blood pressure (BP) was measured using an automated device (model UA-799; A&D), and three readings at the right upper arm were taken while seated (1-minute intervals)(7). We used the mean of the last two readings. Hypertension was defined as systolic BP ≥140 mmHg and/or diastolic BP ≥90 mmHg and/or self-reported use of BP lowering medicine. Body mass index (BMI) was calculated as weight in kg divided by the square of the height in meters. Fasting blood tests were drawn and analysed on the same day. Serum-total cholesterol was analysed by enzymatic colorimetric method, and haemoglobin A1C (HbA1C) was measured by automated HPLC method (Bio-Rad Laboratories, Munich, Germany). HbA1C ≥6.5% (≥48 mmol/mol) and/or fasting glucose ≥7.0 mmol/L was defined as diabetes. An enzymatic method was used to analyse serum creatinine (standardised against isotope dilution mass spectroscopy, CREA plus, Roche Diagnostics, GmbH, Mannheim, Germany). Estimated GFR (eGFR) values were

calculated using the CKD Epidemiology Collaboration (CKD-EPI) equations, which include measurements based on serum creatinine (eGFRcre), cystatin C (eGFRcys), and the combined creatinine-cystatin equation (eGFRcrecys) (8, 9).

Statistical analysis

Study characteristics were listed as means (standard deviation, SD) if normally distributed, or as medians (IQR) in case of a skewed distribution. Categorical data were listed as number (%). Log2 transformation (doubling) of the urinary biomarkers was performed due to skewed data.

To assess differences within characteristics at baseline and between baseline and follow-up, independent/paired sample t-tests and Wilcoxon rank-sum test were used for continuous data, and McNemar's test and chi-squared test were applied for categorical data. To test for linear trends across quartiles of UOCR, linear-by-linear and Cochran-Armitage tests were used for continuous and categorical data, respectively. Spearman's R was used to test correlations between variables.

To investigate associations between the urinary markers and the annual change in GFR, we used all available GFR measurements in a linear mixed model with random intercepts and slopes for time. The interaction coefficients between time and the biomarkers represent the association with GFR change rates. A negative coefficient signifies an association between a higher marker level and a steeper GFR decline. Using GFR slope as a surrogate endpoint in a population with high GFR has been found to be precise and closely associated with the risk of end-stage renal disease and mortality (10-12). A declining GFR slope captures a subtle but consistent loss of kidney function even in the normal range and is therefore more sensitive to early damage. Using multivariable logistic regression, we calculated the odds ratio (OR) for accelerated GFR decline, defined as the 10% of all subjects with the steepest GFR decline slope. There is no consensus on how to define accelerated GFR decline. The 2012 Kidney Disease Improving Global Outcome (KDIGO) CKD Guideline suggested a definition of ≥5 ml/min/1.73 m^2 per year in patients with CKD (13), whereas several studies have used ≥3 ml/min/1.73 m^2 per year (14, 15). In a healthy general population GFR decline is generally slow, and therefore we chose the 10-percentile of the present population as a cut-off . The slope was calculated as described above in a linear mixed model and adjusted for sex, age, and CKD risk factors at baseline: hypertension, BMI, fasting glucose, and current smoking (16). The 208 persons with only one GFR value (baseline GFR) were also included because the linear mixed model imputes a GFR slope for these persons.

We ran four different models; Model 1 was a crude (unadjusted) model. In Model 2, we adjusted for sex, age, and baseline GFR. In Model 3, the same covariates were assessed as in Model 2 plus the urinary biomarkers, and in Model 4, we added the following cardiovascular risk factors: hypertension, cigarettes smoked per day, glucose, BMI, and cholesterol. In different models, the urinary markers were entered either as log2-transformed continuous variables or dichotomized as above or equal to/below the median. In the logistic regression analysis, we added urinary markers as described and categorised them into quartiles, with

the first quartile as a reference. The likelihood ratio test (LRT) was used to assess whether adding the quartile-based model significantly improved the model indicating non-linearity. Logistic regression analyses with restricted cubic splines with three knots were used to visualize the association of UOCR with the risk for accelerated GFR decline. In clinical settings, GFR is usually estimated rather than measured. Therefore, in sensitivity analyses, we ran similar models using eGFRcrecys as the dependent variable.

We tested for interactions between urinary markers and sex in all the regression models. In order to assess the improvement in risk prediction for accelerated GFR decline in models with and without UOCR, we calculated the area under the receiver-operating characteristics (ROC) curve (AUC) for the nested logistic regression model utilising the likelihood ratio test. We adjusted for the variables included in the Kidney Failure Risk Equation (KFRE); age, sex, baseline GFR, UACR (17), and in the comprehensive model we further included hypertension, BMI current smoking, cholesterol and fasting glucose as previously used in publications from the RENIS studies .

A p-value < 0.05 was considered significant. We employed the statistical software STATA 18.0 (StataCorp, College Station, TX, USA) and R version 3.5.1 *(*R Foundation for Statistical Computing, Vienna, Austria).

Results

Cohort characteristics

A total of 1,589 participants providing 4,323 GFR measurements were included, with a mean follow-up time of 11.3 (±0.6) years. Fifty-one percent were women, the mean (SD) age was 58.1 (3.8) years, mean (SD) GFR was 93.8 (14.3) and eGFRcrecys 103 (11.4) ml/min/1.73 m². Median (IQR) UOCR was 0.04 (0.02-0.08) mg/mmol, UACR 0.29 (0.16- 0.54) mg/mmol**.** Grouped by quartiles of UOCR, the study participants in the highest quartile had a more adverse metabolic profile with higher BP, BMI and levels of both UOCR and UACR. Men had higher BP, BMI, GFR and UOCR, but lower UACR than women . The participants who did not participate in the last follow-up (RENIS-3, n=441), did not differ significantly from the other participants, except for being older and having marginally higher UOCR and UACR

UOCR was moderately correlated with baseline UACR (r =0.45, p<0.001) and weakly or non-correlated with age and cardiovascular risk factors. The correlation between the concentrations of the urinary markers without adjustment for creatinine (UOE and urinary albumin excretion (UAE)) was similar to the correlations between the creatinine adjusted variables (r=0.48, p<0.001). Neither UOCR nor UACR were correlated to baseline GFR, but each urinary marker was weakly, but significantly correlated to accelerated GFR decline

GFR change rates

The mean (SD) GFR change was -1.07 (0.03) ml/min/1.73 m²/year, calculated using a linear mixed model (a negative rate of change signifies a decline in GFR). A doubling of UOCR was associated with a faster annual GFR decline of -0.06 (-0.10 to -0.01) ml/min/1.73 m^2 per year, p=0.02) when adjusting for age, sex, baseline GFR, and UACR. Similarly, UOCR above compared to equal to/below the median was associated with a steeper annual GFR decline rate of -0.17 (-0.30 to -0.04) mL/min/1.73 m^2, p=0.008. However, in the fully adjusted model, a doubling of UOCR lost statistical significance (p=0.08), but UOCR above the median was still associated with a steeper annual decline in GFR (-0.13 (-0.26 to -0.003) ml/min/1.73 m^2, p=0.04).

Similar analyses were conducted for UACR. However, no significant associations were found with annual change in GFR, neither in crude nor adjusted models.

Accelerated GFR decline

Accelerated GFR decline corresponded to a mean GFR decline ≥1.53 mL/min/1.73 m²/year (n=159). In crude models, higher UOCR was significantly associated with this endpoint when entered as a log2 transformed continuous variable and when categorised into above versus below the median or into quartiles. With the full adjustment, UOCR dichotomised into above vs below median remained significantly associated with accelerated GFR decline (OR 1.52, 95%CI 1.02-2.27, p=0.04). UACR was not associated with an accelerated GFR decline in the crude or fully adjusted model.

Supplementary Figure 2 visualises the non-linear association between UOCR in mg/mmol and the age, sex and baseline GFR adjusted OR for accelerated GFR decline using logistic regression analysis (p=0.01 for non-linearity). There were no significant interactions between the urinary markers (UOCR, UACR) and sex.

To quantitate the contribution of UOCR above vs below median to the prediction of accelerated GFR decline, we analysed the AUC of the ROC curve. In a model adjusted for the same variables as in the KFRE (18), the AUC increased from 0.739 to 0.745 (p=0.002). In the fully adjusted model (including hypertension, BMI, cholesterol, glucose and cigarettes per day), AUC increased from 0.831 to 0.832 (p=0.04).

Sensitivity analysis

Substituting measured GFR for eGFRcrecys as the dependent variable in the linear mixed model analyses with the same covariates as used in the main analyses, yielded similar results, but the UOCR above versus equal to/below the median was not significantly associated with eGFRcrecys decline in the fully adjusted model (p = 0.056). Accelerated eGFR decline was defined as a mean annual decline rate ≥2.05 ml/min/1.73$m^{2.}$ Using logistic regression, significant associations were observed with OR 1.76 (1.20-2.58; p=0.004) for accelerated eGFR decline if UOCR was above versus below/equal to the median.

References

1. Eriksen BO, Mathisen UD, Melsom T, Ingebretsen OC, Jenssen TG, Njolstad I, et al. Cystatin C is not a better estimator of GFR than plasma creatinine in the general population. Kidney international. 2010;78(12):1305-11.
2. Jacobsson L. A method for the calculation of renal clearance based on a single plasma sample. Clinical physiology. 1983;3.
3. Melsom T, Norvik JV, Enoksen IT, Stefansson V, Mathisen UD, Fuskevag OM, et al. Sex Differences in Age-Related Loss of Kidney Function. Journal of the American Society of Nephrology : JASN. 2022;33(10):1891-902.
4. Kustan P, Szirmay B, Horvath-Szalai Z, Ludany A, Lakatos A, Muhl D, et al. Urinary orosomucoid: validation of an automated immune turbidimetric test and its possible clinical use. Biochem Med (Zagreb). 2016;26(3):421-30.
5. Kronborg CS, Allen J, Vittinghus E, Knudsen UB. Pre-symptomatic increase in urine-orosomucoid excretion in pre-eclamptic women. Acta Obstet Gynecol Scand. 2007;86(8):930-7.
6. Vittinghus E. Preanalytical handling of stored urine samples, and measurement of beta 2-microglobulin, orosomucoid, albumin, transferrin and immunoglobulin G in urine by enzyme-linked immunosorbent assays (ELISA). Scand J Clin Lab Invest. 1990;50(8):843-9.
7. Mathisen UD, Melsom T, Ingebretsen OC, Jenssen TG, Njolstad I, Solbu MD, et al. Ambulatory blood pressure is associated with measured glomerular filtration rate in the general middle-aged population. Journal of hypertension. 2012;30(3):497-504.
8. Levey AS, Stevens LA, Schmid CH, Zhang YL, Castro AF, 3rd, Feldman HI, et al. A new equation to estimate glomerular filtration rate. Ann Intern Med. 2009;150(9):604-12.
9. Inker LA, Schmid CH, Tighiouart H, Eckfeldt JH, Feldman HI, Greene T, et al. Estimating glomerular filtration rate from serum creatinine and cystatin C. N Engl J Med. 2012;367(1):20-9.
10. Grams ME, Sang Y, Ballew SH, Matsushita K, Astor BC, Carrero JJ, et al. Evaluating Glomerular Filtration Rate Slope as a Surrogate End Point for ESKD in Clinical Trials: An Individual Participant Meta-Analysis of Observational Data. Journal of the American Society of Nephrology. 2019;30(9):1746-55.
11. Inker LA, Collier W, Greene T, Miao S, Chaudhari J, Appel GB, et al. A meta-analysis of GFR slope as a surrogate endpoint for kidney failure. Nat Med. 2023;29(7):1867-76.
12. Leffondre K, Boucquemont J, Tripepi G, Stel VS, Heinze G, Dunkler D. Analysis of risk factors associated with renal function trajectory over time: a comparison of different statistical approaches. Nephrol Dial Transplant. 2015;30(8):1237-43.
13. Group. KCW. KDIGO 2012 Clinical Practice Guideline for the Evaluation and Management of Chronic Kidney Disease. Kidney Int Suppl. 2013;3(1):1–150. doi:10.1038/kisup.2012.73. 2012.
14. Rifkin DE, Shlipak MG, Katz R, Fried LF, Siscovick D, Chonchol M, et al. Rapid kidney function decline and mortality risk in older adults. Arch Intern Med. 2008;168(20):2212-8.
15. Tsai CW, Ting IW, Yeh HC, Kuo CC. Longitudinal change in estimated GFR among CKD patients: A 10-year follow-up study of an integrated kidney disease care program in Taiwan. PLoS One. 2017;12(4):e0173843.
16. Enoksen IT, Svistounov D, Norvik JV, Stefansson VTN, Solbu MD, Eriksen BO, Melsom T. Serum matrix metalloproteinase 7 and accelerated glomerular filtration rate decline in a general non-diabetic population. Nephrol Dial Transplant. 2022;37(9):1657-67.
17. Tangri N, Kitsios GD, Inker LA, Griffith J, Naimark DM, Walker S, et al. Risk prediction models for patients with chronic kidney disease: a systematic review. Ann Intern Med. 2013;158(8):596-603.
18. Tangri N, Stevens LA, Griffith J, Tighiouart H, Djurdjev O, Naimark D, et al. A predictive model for progression of chronic kidney disease to kidney failure. JAMA. 2011;305(15):1553-9.

Supplementary Table 1. Baseline characteristics of the study population (n=1589)

	Total population n=1589	Q1, n=398 (0.004-0.02)	Q2, n=397 (0.02-0.04)	Q3, n=397 (0.04-0.08)	Q4, n=397 (0.08-6.2)	*p*-value linear trend
**Female, n (%)**	811 (51)	206 (52)	229 (58)	204 (51)	172 (43)	0.01
**Age (years)**	58.1±3.8	57.4±3.9	58.1±3.8	58.2±3.8	58.6±3.7	<0.001
**Systolic blood pressure, mmHg**	129±18	125±16	127±15	131±18	135±19	<0.001
**Diastolic blood pressure, mmHg**	83±10	81±10	83±9	84±10	86±10	<0.001
**Body mass index, kg/m²**	27.2±4.0	26.2±3.6	27.2±3.9	27.7±4.3	27.8±3.9	<0.001
**Cholesterol, mmol/l**	5.6±0.9	5.6±0.9	5.6±1.0	5.7±1.0	5.6±0.9	0.11
**HbA1c, %**	5.5±0.3	5.5±0.3	5.5±0.3	5.5±0.3	5.6±0.3	0.002
**GFR, ml/min/1.73m²**	93.8±14.3	94.7±13.1	94.1±13.4	94.0±15.9	92.6±14.8	0.05
**eGFRcre, ml/min/1.73m²**	94.8±9.5	95.3±8.8	95.2±9.4	94.7±10.1	94.2±9.8	0.08
**eGFRcys, ml/min/1.73m²**	105.4±12.3	107.7±10.6	105.6±12.4	104.9±13.1	103.6±12.8	<0.001
**eGFRcrecys, ml/min/1.73m²**	103.0±11.4	104.5±10.2	103.4±11.4	102.7±12.2	101.5±11.7	0.0002
**UOCR, mg/mmol**	0.04 (0.02,0.08)	0.02 (0.01, 0.02)	0.03 (0.03,0.03)	0.05 (0.04, 0.06)	0.15 (0.10, 0.25)	<0.001
**UACR, mg/mmol**	0.29 (0.16,0.54)	0.21 (0.12,0.36)	0.27 (0.15,0.47)	0.31 (0.18, 0.57)	0.42 (0.21, 0.96)	<0.001
**Current smoking, n (%)**	339 (21)	76 (19)	74 (19)	94 (24)	95 (24)	0.06
**Cigarettes numbers**	11.4±6	11.1±5.6	10.1±5.7	12.1±7.4	12.1±6.2	0.09
**Hypertension, n (%)**	654 (41)	125 (31)	148 (37)	165 (42)	216 (54)	<0.001
**Use of BP lowering drugs (%)**	250 (16)	38 (9.5)	56 (14.1)	66 (16.9)	90 (22)	<0.001
**Use of ACE or ARB n (%)**	158 (10)	28 (7)	27 (7)	46 (12)	57 (14)	<0.001

Values are given as mean (±SD) for normally distributed data and as median (Interquartile Range) for skewed data or as number (percent).

GFR: glomerular filtration rate, measured with iohexol. eGFR $_{cre/cys/crecys}$: estimated GFR based on the CKD-EPI equation for creatinine, cystatin C or both. UOCR: urinary orosomucoid creatinine ratio. UACR: urinary albumin creatinine ratio. Cigarette numbers: If current smokers, the number of cigarettes smoked per day. Hypertension: Systolic blood pressure ≥140mmHg and/or diastolic blood pressure ≥90mmHg, and/or the use of blood pressure lowering medication. ACE: angiotensin converting enzyme. ARB: angiotensin receptor blocker

Supplementary Table 2. Study population characteristics at baseline, lost to follow-up in RENIS-3*

	RENIS-T6, baseline n=1148	Lost to follow up in RENIS-3 n=441	P-value
**Female, n (%)**	584 (51)	227 (51)	0.83
**Age**	57.9±3.8	58.4±3.8	0.01
**Systolic blood pressure, mmHg**	129±17	131±18	0.15
**Diastolic blood pressure, mmHg**	83±10	83±10	0.83
**Body mass index, kg/m²**	27.1±3.8	27.5±4.3	0.07
**GFR, ml/min/1.73m²**	94.2±14.0	92.9±15	0.13
**eGFRcre, ml/min/1.73m²**	94.8±9.3	94.9±10.3	0.77
**eGFRcys, ml/min/1.73m²**	105.9±12.1	104.2±12.8	0.01
**eGFRcrecys, ml/min/1.73m²**	103.2±11.2	102.4±11.9	0.20
**UOCR, mg/mmol**	0.04 (0.02,0.07)	0.04 (0.02,0.10)	0.007
**UACR, mg/mmol**	0.27 (0.15,0.52)	0.32 (0.17,0.65)	0.003
**Current smoking, n (%)**	222 (19)	117 (27)	0.002
**Hypertension, n (%)**	236 (21)	117 (27)	0.01
**Use of ACE or ARB n (%)**	106 (9)	52 (12)	0.13

Values are given as mean (±SD) for normally distributed data, median (Interquartile Range) for skewed data, or number (percent).

GFR: glomerular filtration rate, measured with iohexol. $eGFR_{cre/cys/crecys}$: estimated GFR based on the CKD-EPI equation for creatinine, cystatin C or both.

UOCR: urinary orosomucoid creatinine ratio. UACR: urinary albumin creatinine ratio. Hypertension: Systolic blood pressure ≥140mmHg and/or diastolic blood pressure ≥90mmHg, and/or the use of blood pressure lowering medication. ACE: angiotensin converting enzyme. ARB: angiotensin receptor blocker

***Comparing participants with valid GFR measurements in both baseline and last follow-up versus those at baseline with no measurements in Renis-3

Supplementary Table 3. Prediction of accelerated GFR decline with UOCR above median*

	Model 1	Model 2	Model 3
**AUC without UOCR**	0.739	0.773	0.831
**AUC with UOCR**	0.745, p=0.002	0.778, p=0.001	0.832, p=0.04

Model 1: adjusted for sex, age
Model 2: adjusted for sex, age, GFR, UACR
Model 3: adjusted for sex, age, GFR, UACR, Hypertension, BMI, smoke, glucose, cholesterol
GFR: glomerular filtration rate, measured with iohexol. UOCR: urinary orosomucoid creatinine ratio. UACR: urinary albumin creatinine ratio. Hypertension: Systolic blood pressure ≥140mmHg and/or diastolic blood pressure ≥90mmHg, and/or the use of blood pressure lowering medication
*Accelerated GFR decline defined as annual GFR decline ≥1.53mL/min/1.73m²/year, n=159 (10% steepest decline)
P for the AUC difference with and without UOCR above median

Supplementary Table 4. Correlation between urinary markers and covariates

	UOCR	P-value	UACR	P-value
**UACR**	0.45	<0.001	--	--
**Sex**	0.04	0.14	0.02	0.44
**Age**	0.07	0.01	0.04	0.10
**Systolic blood pressure, mmHg**	0.09	<0.001	0.10	<0.001
**Diastolic blood pressure, mmHg**	0.07	0.01	0.10	<0.001
**Body mass index, kg/m²**	0.06	0.02	0.06	0.01
**Cholesterol mmol/l**	0.02	0.55	0.004	0.87
**HbA1C**	0.05	0.06	0.06	0.02
**GFR ml/min/1.73m²**	-0.03	0.23	-0.01	0.83
**Current smoking**	0.02	0.54	0.11	<0.001
**Hypertension**	0.06	0.01	0.10	<0.001
**ACE or ARB**	0.09	<0.001	0.12	<0.001
**Accelerated GFR decline**	0.08	0.002	0.06	0.02

GFR: glomerular filtration rate, measured with iohexol

UOCR: urinary orosomucoid creatinine ratio. UACR: urinary albumin creatinine ratio. Hypertension: Systolic blood pressure ≥140mmHg and/or diastolic blood pressure ≥90mmHg, and/or the use of blood pressure lowering medication. ACE: angiotensin-converting enzyme. ARB: angiotensin receptor blocker

[a] defined as annual GFR decline ≥1.53 mL/min/1.73m², n=159 (10% with the steepest decline)

Supplementary Table 5. Change in annual eGFRcrecys decline explained by urinary markers during the whole study period

	Doubling (log2) of UOCR		Doubling (log2) of UACR		UOCR above median		UACR above median	
	ml/min/1.73 m^2/year	P-value	ml/min/1.73 m^2/year	P-value	ml/min/1.73 m^2/year	P-value	ml/min/1.73 m^2/year	P-value
**Model 1**	-0.03 (-0.06 to -0.003),	0.03	-0.03 (-0.07 to 0.001),	0.04	-0.11 (-0.19 to -0.02),	0.01	-0.02 (-0.10 to 0.06),	0.62
**Model 2**	-0.04 (-0.07 to -0.01),	0.01	-0.03 (-0.07 to 0.15),	0.06	-0.11 (-0.19 to -0.04),	0.004	-0.01 (-0.10 to 0.07),	0.81
**Model 3**	-0.03 (-0.07 to -0.002),	0.04	-0.02 (-0.06 to 0.01),	0.18	-0.10 (-0.18 to –0.01),	0.026	0.01 (-0.09 to 0.08),	0.99
**Model 4**	-0.03 (-0.06 to 0.0003),	0.07	-0.02 (-0.05 to 0.02),	0.30	-0.08 (-0.17 to 0.002),	0.056	0.006 (-0.08 to 0.09),	0.89

Model 1) Crude, urinary protein (mg/mmol) only

Model 2) adjusted for sex, age and baseline eGFR,

Model 3) adjusted for sex, age, baseline eGFR and UACR/UOCR,

Model 4) as in model 3 and adjusted for hypertension, smoke, glucose, BMI, cholesterol

UOCR: urinary orosomucoid creatinine ratio. UACR: urinary albumin creatinine ratio. Hypertension: Systolic blood pressure ≥140mmHg and/or diastolic blood pressure ≥90mmHg, and/or the use of blood pressure lowering medication.

eGFRcrecys: estimated glomerular filtration based on the CKD_EPI equation for creatinine and cystatin C.

Supplementary Table . OR for accelerated eGFRcrecys decline*

	UOCR			UACR		
**Model 1)**	**OR**	**95% CI**	***p*-value**	**OR**	**95% CI**	***p*-value**
Doubling of urinary protein (log2)	**1.28**	**1.16- 1.43**	**<0.001**	**1.29**	**1.15-1.44**	**<0.001**
First quartile	*Ref.*			*Ref.*		
Second quartile	**1.56**	**0.86-2.82**	**1.47**	**2.89**	**1.67-5.00**	**<0.001**
Third quartile	**2.86**	**1.65-4.93**	**<0.001**	**1.66**	**0.93-2.98**	**0.09**
Fourth quartile	**3.23**	**1.89-5.53**	**<0.001**	**3.23**	**1.88-5.53**	**<0.001**
Below median	*Ref.*			*Ref.*		
Above median	**2.37**	**1.67-3.38**	**<0.001**	**1.27**	**0.92-1.77**	**0.15**
**Model 2)**						
Doubling of urinary protein (log2)	**1.18**	**1.05-1.31**	**0.004**	**1.26**	**1.12-1.43**	**<0.001**
First quartile	*Ref.*			*Ref.*		
Second quartile	**1.37**	**0.74-2.53**	**0.32**	**3.34**	**1.86-5.99**	**<0.001**
Third quartile	**2.29**	**1.30-4.05**	**0.004**	**1.81**	**0.97-3.36**	**0.06**
Fourth quartile	**2.28**	**1.30-3.98**	**0.004**	**3.61**	**2.03-6.39**	**<0.001**
Below median	*Ref.*			*Ref.*		
Above median	**1.91**	**1.32-2.77**	**0.001**	**1.28**	**0.90-1.82**	**0.16**
**Model 3)**						
Doubling of urinary protein (log2)	**1.15**	**1.02-1.30**	**0.024**	**1.33**	**1.12-1.58**	**0.001**
First quartile	*Ref.*			*Ref.*		
Second quartile	**1.36**	**0.74-2.52**	**0.33**	**3.32**	**1.85-5.95**	**<0.001**
Third quartile	**2.27**	**1.28-4.01**	**0.005**	**1.79**	**0.96-3.31**	**0.067**
Fourth quartile	**2.12**	**0.01-1.20**	**0.010**	**3.38**	**1.86-6.14**	**<0.001**
Below median	*Ref.*			*Ref.*		
Above median	**1.84**	**1.26-2.68**	**0.001**	**1.18**	**0.82-1.71**	**0.36**
**Model 4)**						
Doubling of urinary protein (log2)	**1.13**	**1.00-1.28**	**0.053**	**1.32**	**1.11-1.57**	**0.001**
First quartile	*Ref.*			*Ref.*		
Second quartile	**1.37**	**0.74-2.55**	**0.32**	**3.41**	**1.88-6.18**	**<0.001**
Third quartile	**2.24**	**1.26-4.00**	**0.32**	**1.89**	**1.01-3.54**	**0.047**
Fourth quartile	**1.99**	**1.12-3.55**	**0.02**	**3.42**	**1.87-6.27**	**<0.001**
Below median	*Ref.*			*Ref.*		
Above median	**1.76**	**1.20-2.58**	**0.004**	**1.21**	**0.84-1.74**	**0.32**

Model 1) crude, urinary protein (mg/mmol) only, Model 2) adjusted for sex, age and baseline GFR, Model 3) adjusted for sex, age, baseline GFR and UACR/UOCR,Model 4) as in model 3 and adjusted for hypertension, smoke, glucose, BMI, cholesterol. *Defined as the 10% with steepest decline, annual eGFR decline ≥2.05 ml/min/1.73m^{2}. eGFRcrecys: estimated glomerular filtration rate based on the CKD-EPI equation for both creatinine and cystatin C. UOCR: urinary orosomucoid creatinine ratio. UACR: urinary albumin creatinine ratio. Hypertension: Systolic blood pressure ≥140mmHg and/or diastolic blood pressure ≥90mmHg, and/or the use of blood pressure lowering medication

Supplementary Figure 1. Flowchart of the study population

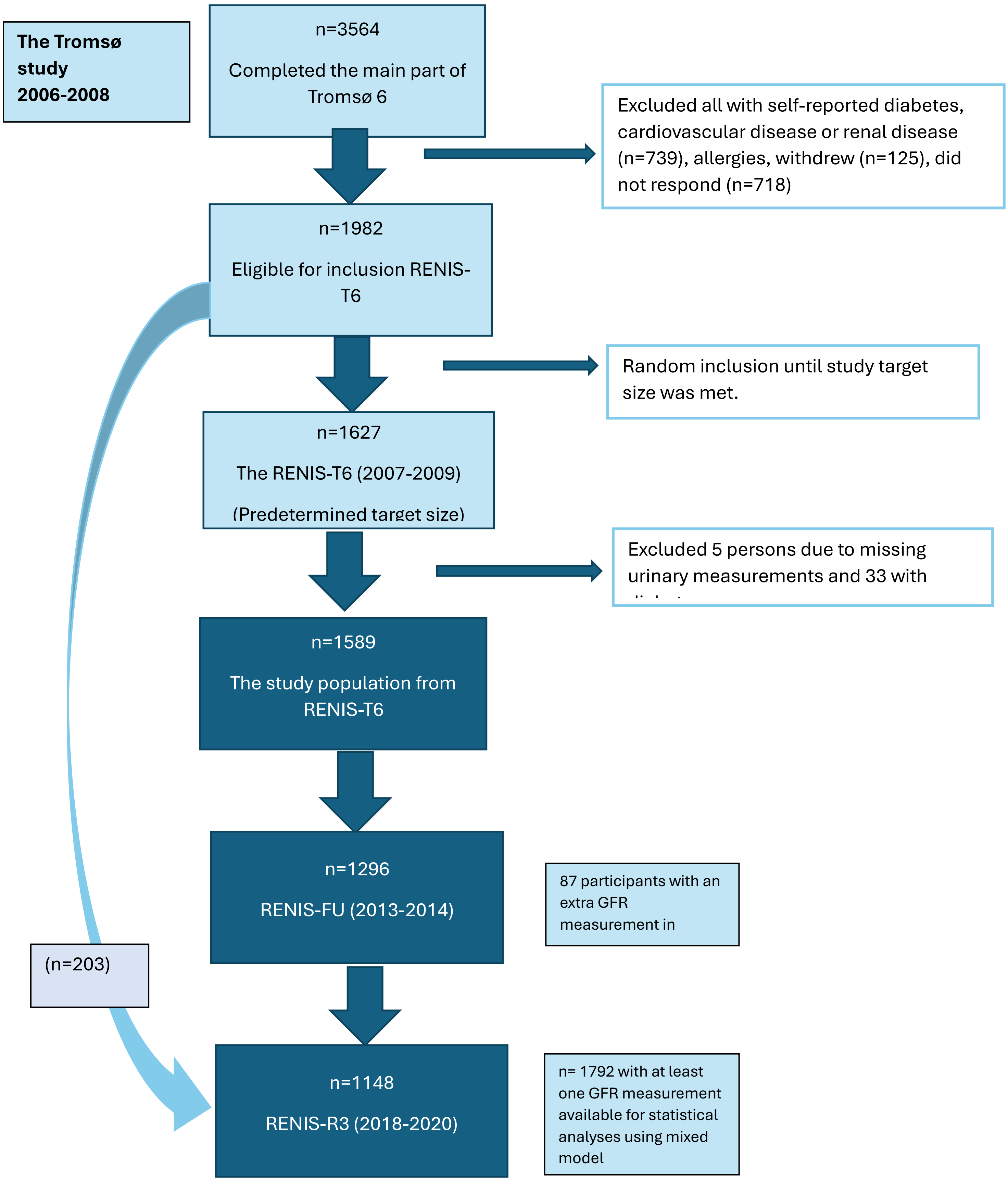

Supplementary Figure 2. The non-linear association between UOCR and OR for accelerated GFR decline

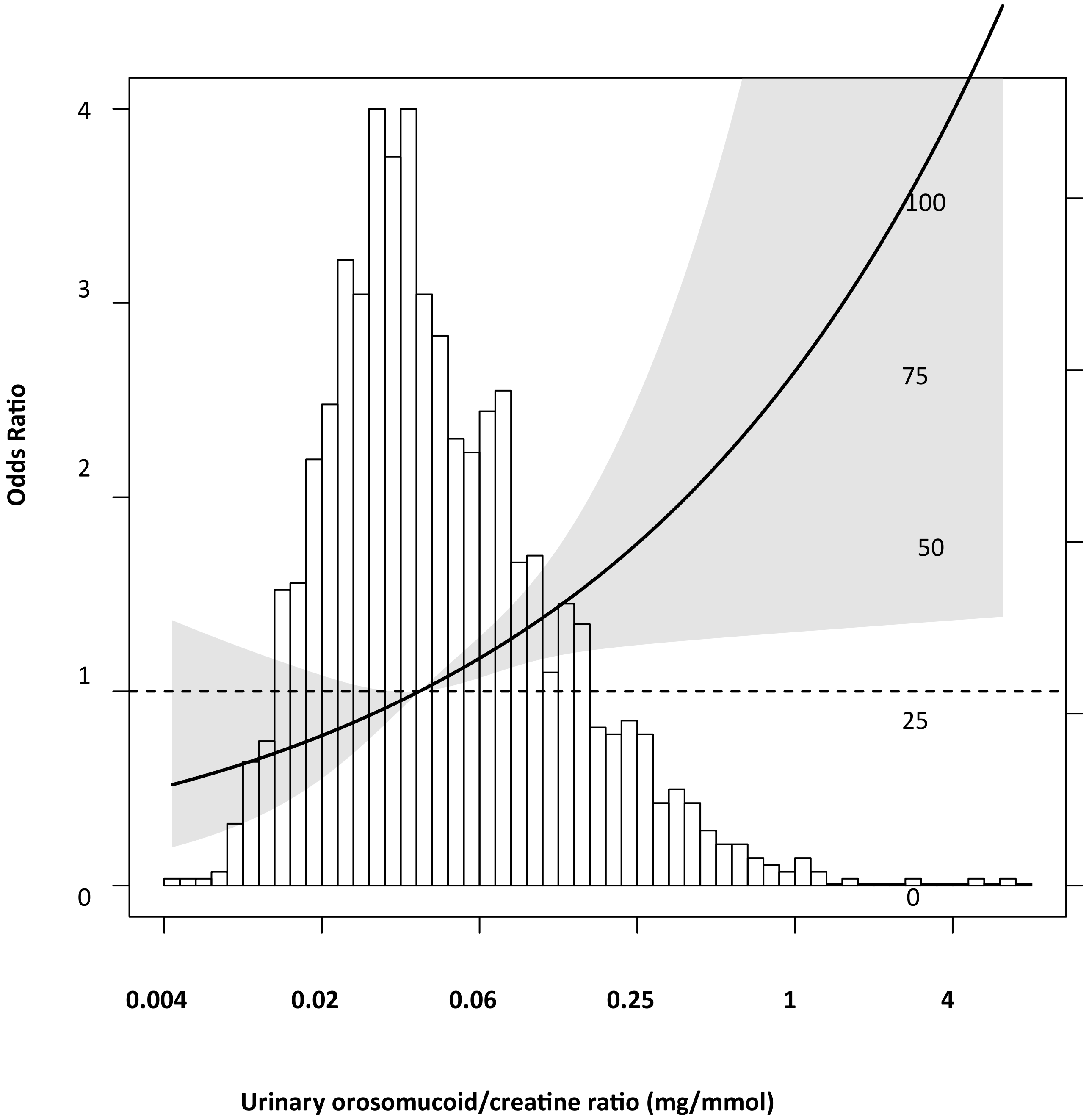

Supplement: gfaf113_Supplemental_File [file gfaf113_supplemental_file.pdf]
